# New Insights on the Management of Wildlife Diseases Using Multi-State Recapture Models: The Case of Classical Swine Fever in Wild Boar

**DOI:** 10.1371/journal.pone.0024257

**Published:** 2011-09-22

**Authors:** Sophie Rossi, Carole Toigo, Jean Hars, Françoise Pol, Jean-Luc Hamann, Klaus Depner, Marie-Frederique Le Potier

**Affiliations:** 1 Unité Sanitaire de la Faune, Office National de la Chasse et de la Faune Sauvage, Gap, France; 2 CNERA Faune de Montagne, Office National de la Chasse et de la Faune Sauvage, Gières, France; 3 Laboratoire National de Référence pour les Pestes Porcines, French Agency for Food Envionmental and Occupational Health and Safety, Ploufragan, France; 4 CNERA Cervidés-Sanglier, Office National de la Chasse et de la Faune Sauvage, Petite Pierre, France; 5 Institute of Diagnostic Virology, Friedrich-Loeffler-Institut, Greifswald-Insel Riems, Germany; Massey University, New Zealand

## Abstract

**Background:**

The understanding of host-parasite systems in wildlife is of increasing interest in relation to the risk of emerging diseases in livestock and humans. In this respect, many efforts have been dedicated to controlling classical swine fever (CSF) in the European Wild Boar. But CSF eradication has not always been achieved even though vaccination has been implemented at a large-scale. Piglets have been assumed to be the main cause of CSF persistence in the wild since they appeared to be more often infected and less often immune than older animals. However, this assumption emerged from laboratory trials or cross-sectional surveys based on the hunting bags.

**Methodology/Principal Findings:**

In the present paper we conducted a capture-mark-recapture study in free-ranging wild boar piglets that experienced both CSF infection and vaccination under natural conditions. We used multi-state capture recapture models to estimate the immunization and infection rates, and their variations according to the periods with or without vaccination. According to the model prediction, 80% of the infected piglets did not survive more than two weeks, while the other 20% quickly recovered. The probability of becoming immune did not increase significantly during the summer vaccination sessions, and the proportion of immune piglets was not higher after the autumn vaccination.

**Conclusions/Significance:**

Given the high lethality of CSF in piglets highlighted in our study, we consider unlikely that piglets could maintain the chain of CSF virus transmission. Our study also revealed the low efficacy of vaccination in piglets in summer and autumn, possibly due to the low palatability of baits to that age class, but also to the competition between baits and alternative food sources. Based on this new information, we discuss the prospects for the improvement of CSF control and the interest of the capture-recapture approach for improving the understanding of wildlife diseases.

## Introduction

Understanding the mechanisms of disease dynamics in wildlife populations is of increasing interest in relation to the risk of emerging diseases in livestock and humans [1]. In this respect, wild boar (*Sus scrofa* sp.) have been the subject of much work as the increase in their numbers throughout Europe has led to an increasing risk of disease emergence, persistence and transmission to other species [2], [3]. The classical swine fever (CSF) virus is one of the persisting pathogens observed among European wild boar populations [4], [5], [6], [7], [8] and represents a major source of disease for the domestic pig, with potentially substantial economic consequences [9]. The management of wild CSF outbreaks is mandatory in the European Union (Council Directive 2001/89 EC). Oral vaccination is considered as the main tool for controlling CSF in the wild [10], [11]. However, infection has sometimes persisted for years or re-emerged despite a huge vaccination effort [11]. Accordingly, a better understanding of CSF dynamics and vaccination effect is required.

Because they appeared to be more often infected and less often immune than older animals, the young wild boar have been assumed to be important virus carriers, which had to be either destroyed or vaccinated in their early life [4], [6], [12]. However, hypotheses on the role of piglets and their capacity to eat the vaccine-baits have derived from experiments conducted under laboratory conditions [13], [14], [15], [16] or from the percentages of immune/infected individuals observed in the hunting bags [4], [12], [7]. The interpretation of the effect of vaccination using hunting data is particularly questionable because sampling bias never can be ruled out from cross-sectional studies. Moreover vaccination and infection induce the same antibody reaction: a seropositive individual could either have been vaccinated or have been infected and have recovered [11]. To our knowledge, longitudinal studies aiming to describe the individual outcome of infection and immunization have never been performed in the wild.

The present paper investigates individual histories in free-ranging wild boar that were captured, marked and recaptured. The study was performed in an area where a natural outbreak of CSF occurred and where vaccination was implemented [17]. We targeted 2–7 month old piglets, which were supposedly the most at risk of being infected [11] and which could be recaptured more frequently than older individuals [18]. A multi-state capture-mark-recapture (CMR) modelling approach was used to estimate the probability of becoming infected and of becoming immune during and outside of the vaccination periods.

Using this approach, we first described the outcome of infection (duration/mortality) in piglets in the wild to discuss their capacity to maintain the chain of transmission. Secondly, we determined the effect of vaccination in piglets and the prospects for improving CSF control in wild populations.

## Materials and Methods

### 1. Study area

**Figure 1:**
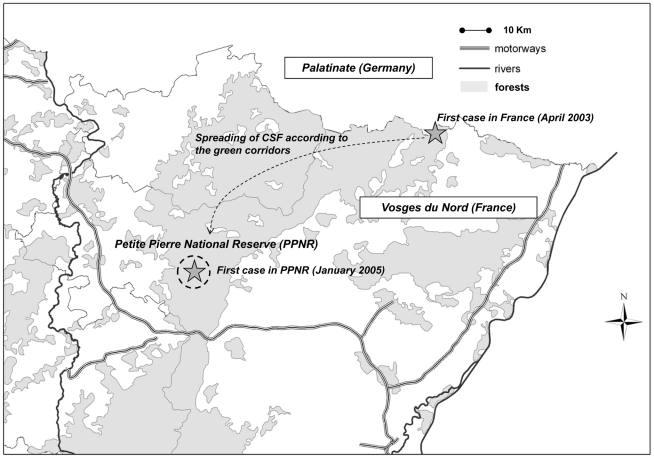
The study area (Petite Pierre National Reserve) is located in the Vosges Mountains and had been infected from January 2005 to November 2006.

The study was conducted in the Petite Pierre National Reserve (PPNR), north-eastern France (48.5°N,7°E) [19], [20]. The PPNR is an unfenced 2,800 ha area located in the Vosges Mountains, *i.e.*, a continuous forested area (>3,000 km^2^) inhabited by a wild boar metapopulation where CSF virus has been demonstrated to circulate (Fig. 1) [17], [21]. Two CSF waves have been documented in the Vosges Mountains: a first wave during the 1990s and a second wave from 2003 to 2007 [17], [21] (Fig. 1). During the second wave, the CSF virus has been observed in the PPNR from January 2005 up to November 2006. An approximate number of 400 wild boar (before the hunting period and after births) may be estimated, considering that 150 wild boar are hunted on average each year in the PPNR, and assuming that each individual wild boar has the same probability of being shot-dead as in the area studied by Toigo et al. (2008) [22].

### 2. Wild boar sampling

**Figure 2:**
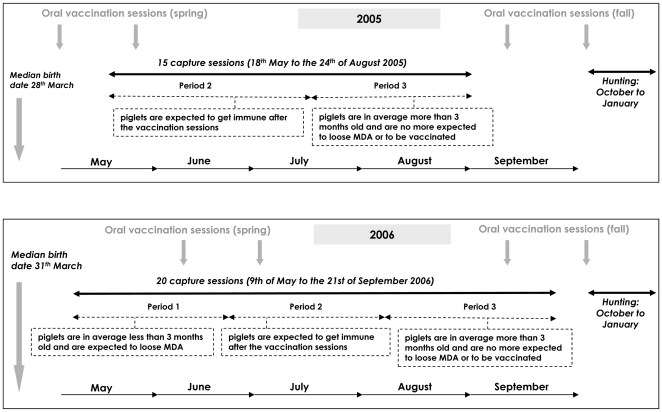
Time periods defined according to the vaccination sessions and the age of piglets.

Captures were performed once a week from 18^th^ May to 24^th^ August 2005 and from 9^th^ May to 21^st^ September 2006 (Fig. 2), using box traps specifically adapted for catching piglets [23]. In order to maximize the probability of capturing different individuals, 11 traps were set in different valleys. Blood samples were taken for serological and virological examination. Each trapped animal was marked with ear-tags to allow individual identification and was released immediately after handling without anaesthesia.

All wild boar killed by hunters in the study area and its surroundings were compulsorily subjected to serological and virological examinations [17], [24], [25]. We focused our analysis on individuals less than one year old shot in November (i.e., just after the autumn vaccination sessions). Individuals were aged from tooth eruption or body weight [19], [18], with carcasses of less than 30 kg assumed to be less than 1 year old.

### 3. Diagnosis of disease status

For antibody examination, commercially available ELISA kits (Herdcheck CSFV Antibody test kit or CHEKIT CSF SERO Antibody, both distributed by IDEXX® and having the same sensitivity) were used according to the manufacturer's instructions. For virological examination, the CSF virus genome was first amplified by real-time polymerase chain reaction (r-RT-PCR) using a commercial kit (TAQVET PPC® or ADIAVET CSF®) according to manufacturer's instructions [26], [27], [28] To confirm the viropositive result, virus isolation or sequencing were performed on the PCR positive samples at the French Reference Laboratory for CSF (ANSES) according to the EU-Diagnostic Manual for CSF (Decision 2002/106/EC).

### 4. Oral vaccination

Oral vaccination had been implemented in the study area since February 2005 according to the protocol recommended by [29], i.e., three 1-month interval double distributions of vaccine-baits in spring, autumn and winter. In 2005, distributions were conducted on the 12^th^ February/12^th^ March (winter), on the 7^th^ May/4^th^ June (spring) and on the 27^th^ August/24^th^ September (autumn). In 2006, distributions were conducted on the 25^th^ March/22^nd^ April (winter), on the 3^rd^ June/1^st^ July (summer) and on the 9^th^ September/7^th^ October (autumn) (Fig. 2). Vaccination was expected to influence the proportion of immune individuals 2 to 4 weeks after each vaccination because baits consumption occurs within a few days of deployment [30], [31] and 2 to 4 weeks are required for seroconversion [32]. To estimate the birth dates of piglets in 2005 and 2006, we estimated the age of foeti from females hunted from November 2004 to January 2005 and from November 2005 to January 2006 (Rossi unpublished data). For each litter, we estimated the birth date using the growth curve of Hugget and Widdas [33]. The median date of birth was estimated as the 28^th^ March in 2005 and the 31^st^ March in 2006. Accordingly, most piglets were younger than 4.5 months during winter and spring vaccinations, but older during autumn vaccinations. According to laboratory experiments [15] piglets are likely to eat baits from the age of 4.5 months; the probability of becoming immune was thus expected to be much higher after the autumn than after the summer vaccination session.

### 5. Individual disease states

Animals were classified into three disease states:

- SU: susceptible individuals that may become infected (i.e., seronegative and vironegative individuals).
- INF: infected individuals (i.e., viropositive individuals that were either seropositive or seronegative).
- IM: immune individuals protected at least partially by antibodies against infection (i.e., seropositive and vironegative individuals).

### 6. Seroprevalence

To test the effect of autumn vaccination on immunity in piglets, we compared the proportion of immune individuals (seroprevalence) among those captured before vaccination (August) and those shot after vaccination (November). For this purpose we used only the last observation for piglets captured in August and we tested the difference between these two proportions using the normal approximation. The statistical analyses were performed using R 2.7.2 (the R foundation for statistical computing 2008, available at http://www.r-project.org/).

### 7. Multi-state capture-mark-recapture approach

#### 7.1 The Jolly movement model (JMV)

**Figure 3:**
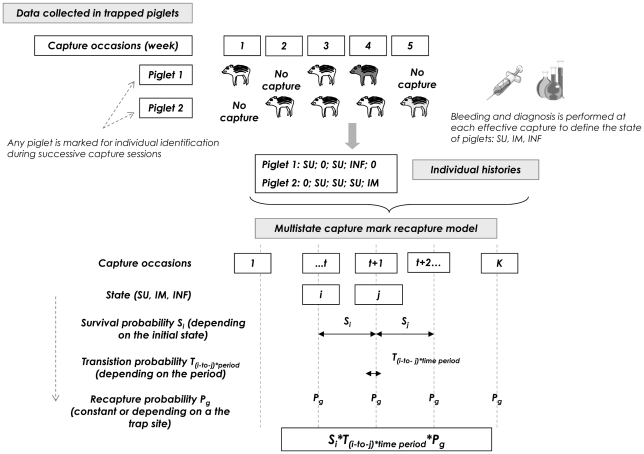
Survival and transition histories of piglets. Individuals are classified into 3 states: susceptible (SU), immune (IM), infected (INF). Transitions are possible between all states except from INF to SU (fixed at zero).

In wildlife ecology, capture-mark-recapture (CMR) modelling has been developed for estimating the survival rate in animals that have been marked and recaptured (or resighted) from time to time and for which the date of death is unknown [34]. The data collected according to CMR approaches for one individual (individual histories) can be summarized as a series of ones and zeros, animals being recaptured or not recaptured during a series of capture sessions. Specific multiplicative multinomial models have been developed for estimating separately the probability of survival and of recapture between two capture events for a group of individuals. These models have been progressively generalized to take into account differences in capture and survival rates over time or among different groups [34]. Then, multistate CMR models were developed to take into account the fact that individuals could also experience different “states” from time to time. In multistate CMR approaches, the individual history is a series of zeros (no successful recapture) and categorical values depending on the state of each individual observed at each effective capture (Fig. 3). In order to take into account possible “movement” between states over time, the Jolly movement model (JMV) has been developed for estimating the probability of transition from one state to another between two capture sessions [35]. According to the JMV model (Fig. 3), the recapture of one individual at time t+1 and in state j, given that this animal was captured at time t and in state i, depends on three probabilities: first, the probability of survival depending on the initial state i, then the probability of transition between states i and j (conditionally to the survival), and lastly, the probability of being recaptured that may either be constant or dependent on time, groups or states. The model parameters are estimated using an iterative process between the model and the observed data, according to the principle of maximum likelihood [35].

#### 7.2 Application of the JMV model to epidemiology

**Figure 4:**
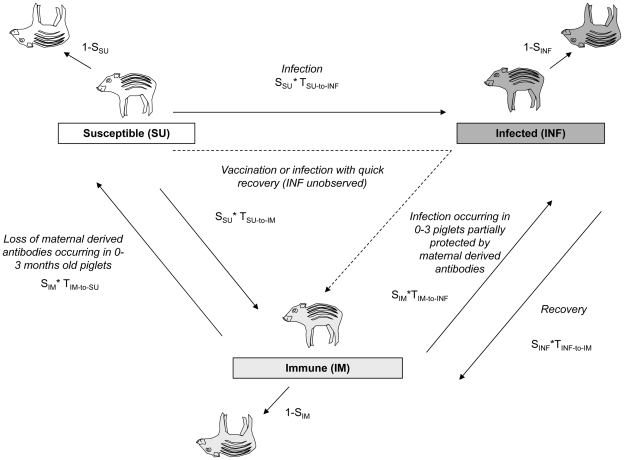
Construction of the Jolly Movement model (JMV) using the individual capture histories of piglets.

In the present study, the JMV model was used to estimate the survival and the probability for any piglet to move between the three states previously defined (Fig. 4). We were particularly interested in estimating the immunization and infection rates classically described in epidemiological models, corresponding to the probabilities for any susceptible piglet to become immune or infected (T_SU-to-IM_ or T_SU-to-INF_) between two captures sessions [36], [37], [38] (Fig. 4). The captures were performed weekly to take into account the virus dynamics, our recapture capacity and the welfare of wild piglets (maximum of one bleeding per animal and per week). All the “movements” were considered as possible, except from state INF to state SU because infected individuals either die or recover but never move back to the susceptible state [32]. Since we captured piglets less than 7 months old, antibodies had three potential origins: natural infection, vaccination or maternally derived antibodies. Differentiation between antibody origin on a single blood sample was not possible (Pol unpublished data) so we explored the variation of the immunization rate according to the time period. In 0–3 months old piglets, maternally derived antibodies (MDA) are gradually disappearing [39]. Contrary to MDA, the immunity induced by vaccination or natural infection (active immunity) is considered lifelong whatever the age of the piglets [32]. As a result of oral vaccination, the probability of becoming immune was supposed to increase during the vaccination periods (i.e., 2 to 4 weeks after each vaccination session), while active immunity was expected to occur at any time due to infection. We also considered that susceptible animals becoming immune out of the vaccination periods could have been infected for a short time but not observed during this period (INF_unobserved_) (Fig. 4). To address these biological hypotheses we explored the variations of the immunization rate according to three time periods (Fig. 2):

- Period 1: during the period when piglets were on average 0–3 months old and when vaccination was not performed, the probability to lose antibodies (passively transmitted by the mother) was expected to be higher than during the two other periods,
- Period 2: during the vaccination sessions, whatever the age the piglets, the probability of acquiring antibodies (after consuming the oral vaccine) was expected to be higher than during the two other periods,
- Period 3: during the period when they were on average more than 3 months old and when vaccination was not performed, piglets were no longer expected to lose or acquire antibodies, except due to some unobserved short-term non-lethal infection.

We conducted separate analyses for 2005 and 2006, because different individuals were concerned. In order to detect possible infringement of the model hypotheses (recapture heterogeneity between individuals or over time) we performed goodness-of-fit (GOF) tests of the fully time-dependent Jolly Move model (JMV), using the program U-Care 2.2.5 [40] (available at http://www.cefe.cnrs.fr). Then, taking into account the GOF analysis, the JMV modelling was performed using M-SURGE 8 [35], [41] (available at http://www.cefe.cnrs.fr). We compared the models, either assuming a constant survival or a survival depending on the state. Survival was expected to be lower in infected than in uninfected individuals owing to the potential lethal effect of CSF virus [16], [42]. Survival might also be lower in susceptible than in immune animals due to the occurrence of lethal-acute infections in piglets that were thus no longer captured. Starting with the best model regarding survival, we compared the models, assuming that transition probabilities were either dependent or independent of the time periods previously defined. Given that we aimed to test the effect of several covariables (state, time periods) on the survival and the transition probabilities, which enhanced the risk of type I error, and that the models we compared were not all nested, model selection was based on the Akaike Information Criterion corrected for small sample size and adjusted for over-dispersion (QAICc) [43]. When the difference in QAICc was less than 2, the most parsimonious model was selected [43]. Once the model selection was achieved, significant differences between specific parameters of the “best model” were tested using Wald tests at the threshold of p≤0.05 using M-SURGE 8 [41], [43].

#### 7.3 Models' predictions

We used the parameters estimated from the “best model” to predict the variation in the numbers of SU, INF, and IM individuals over time and the respective proportions of lethal-chronic (i.e. infected animals that die later than 4 weeks post-infection), lethal-acute (i.e. infected animals that die before 4 weeks post-infection) or transient infections (i.e. infected animals that recover before 4 weeks post-infection) such as defined by Kramer-Schadt et al. [44]. Initial proportions of SU, INF, and IM used in simulations were those observed at first capture, and the initial number of piglets was arbitrarily fixed at 1000 to scale the results.

## Results

### 1. Capture and hunting data

From May to August 2005, 116 piglets were captured between one and 14 times, among which 21 were infected. Among these 21 piglets, none was captured and identified as infected in more than 2 consecutive weeks: they were subsequently either captured and recorded as immune or not recaptured. From May to September 2006, 218 piglets were captured once to 17 times, among which none was infected. In November 2005 and in November 2006 we sampled 49 and 76 hunted piglets (7–10 months old), respectively.

### 2. Seroprevalence

In 2005 and 2006, the mean proportion of seropositive piglets (P) was not significantly higher in piglets shot in November (P_November2005_ = 0.571, n = 49, σ = 0.071; P_November2006_ = 0.276, n = 76, σ = 0.051) than in those captured in August (P_August2005_ = 0.524, n = 24, σ = 0.102; P_August2006_ = 0.426, n = 94, σ = 0.051) (χ^2^_2005_ = 0.190, p = 0.430; χ^2^_2006_ = 1.31, p = 0.096). Hence there was no evidence that the vaccination performed in autumn led to the expected increase in immunity in either year of the study.

### 3. Goodness of fit of the JMV model

Capture transience, corresponding to animals captured only once, was detected in both 2005 (χ^2^ = 33.5, df = 14, P = 0.002) and 2006 (χ^2^ = 70.8, df = 20, P<0.001). This is not surprising because the study area was not fenced and was not large enough to include the home ranges of captured wild boar, and many animals could potentially be captured once while dispersing or at the edge of their home range [45]. Capture transience is an infringement of the assumptions of the JMV model and generates bias in the estimation of survival [46]. To avoid this bias, we removed the first capture from all life histories [47] so that the analyses were finally conducted on 72 and 146 individuals in 2005 and 2006, respectively. We detected no trap-dependence in 2005 (χ^2^ = 2.832, df = 2, p = 0.243), but positive trap-dependence in 2006 [46] (χ^2^ = 42.5, df = 17, p = 0.001) indicating that individuals captured on one occasion were more likely to be captured on the following occasion than others. The trap site location and the social structure of wild boar may have generated this recapture heterogeneity because each trap concerned different family groups, some of them being trap-happy. We overcame this trap-dependence bias by introducing the effect of trap site (n = 11) in the analysis of recapture probability in 2006.

### 4. Selection of CMR models

In 2005, we observed all three disease states previously defined. In 2006 however, only states SU and IM were represented. The number of parameters and QAIC_c_ of the models are detailed in Table 1.

- Capture probabilities: In 2005, the average recapture probability was 0.602 (se = 0.037) and in 2006, it varied from 0.096 (se = 0.039) to 0.609 (se = 0.041) depending on the trap site.
- Survival probabilities: In 2005, survival rate was related to disease state (models with state-dependent survival had lower QAIC_c_ than models with constant survival). The model with different survival rates between the three disease states and the model including only a difference in survival between infected and uninfected individuals had similar QAIC_c_. According to the principle of parsimony, we retained the latter. Survival was significantly lower in infected (S_INF-2005_ = 0.330, se = 0.176) than in susceptible or immune piglets (S_SU-2005_ = 0.871, se = 0.023) (W = 3.05, p = 0.001), confirming the high lethality of CSF infection in that age class. In 2006, survival differed between disease states (the model with state-dependent survival having a lower QAIC_c_ than that with constant survival): susceptible individuals had a significantly lower survival rate (S_SU-2006_ = 0.848, se = 0.026) than immune piglets (S_IM-2006_ = 0.987, se = 0.007) (W = 5.16, p<0.001), possibly because we failed to recapture some lethally infected piglets.
- Transition probabilities: In both 2005 and 2006, the probability of transition depended on the time period (models with time-dependent transitions having lower QAIC_c_ than models with a constant rate of transition, Table 1). In 2005, the probability of antibody loss was higher during the period 2 (T_IMtoSU-period2_ = 0.177; se = 0.081) than during the period 3 (T_IMtoSU-period3_ = 0), corresponding to the expected loss of MDA in 0–3 months old piglets. In 2006, on the contrary, the probability of antibody loss was null during the period 1, (i.e., when piglets were <3 month of age) and was lower during the period 2 (T_IMtoSU-period2_ = 0.094; se = 0.017) compared to the period 3 (T_IMtoSU-period2_ = 0.252; se = 0.036) (W = 3.97, p<0.001). This observation possibly arises because of higher antibody rates in mothers' colostrums in 2006 compared to 2005. Considering the individual histories, we observed that antibody loss occurred mainly when the piglets were in average 4–5 months old. The antibody loss could also be lower during the period 2 compared to the period 3 due to vaccination. But during both study years, we detected no effect of the vaccination period on the probability of becoming immune (T_SUtoIM_) (models with time-dependent transitions having a higher QAIC_c_ than models with a constant rate of transition, Table 1) suggesting that few piglets acquired antibodies consecutive to the summer vaccination sessions. In 2005, both susceptible and immune animals became infected. The probability of becoming infected tended to be lower in immune (T_IMtoINF-2005_ = 0.026; se = 0.019) than in susceptible animals (T_SUtoINF-2005_ = 0.083; se = 0.030) although this effect was only marginally statistically significant (W = 1.61, p = 0.055). This trend is consistent with the partial protection provided by MDA during the first months of life [14]. The estimation of the recovery rate (i.e., the probability of moving from INF to IM) was not accurately estimated because too few infected piglets were recaptured later as immune, most of them remaining unseen after one to two weeks after the first detection of infection. The probability of becoming infected (T_SUtoINF_, T_IMtoINF_) or recovered (T_INFtoIM_) was not significantly different among the periods (models with time-dependent transitions having a higher QAIC_c_ than models with a constant rate of transition, Table 1).

**Table 1:** Selection of the JMV models according to the QAICc values.

Model id	2005/models	QAICc
M1	P_(constant)_,S_(SU,IM,INF)_,T_from(SU,IM,INF)to(SU,IM,INF)*period(2,3)_	658.03
M2	P_(constant)_,S_(SU or IM,INF)_,T_from(SU,IM,INF)to(SU,IM,INF)*period(2,3)_	655.98
M3	P_(constant)_,S_(SU or IM or INF)_, T_from(SU,IM,INF)to(SU,IM,INF)+_ T_from(SU,IM,INF)to(SU,IM,INF) *period(2,3)_	665.54
M4	P_(constant)_,S_(SU or IM or INF)_, T_from(SU,IM,INF)to(SU,IM,INF)+_ (T_from(SU,IM)to(SU,IM) and_ T_from(IM)to(INF)_)_*period(2,3)_	654.00
M5	P_(constant)_,S_(SU or IM or INF)_, T_from(SU,IM,INF)to(SU,IM,INF)+_ (T_from(SU,IM)to(SU,IM)_)_*period(2,3)_	652.46
*M6*	P_(constant)_,S_(SU or IM or INF)_, T_from(SU,IM,INF)to(SU,IM,INF)+_ (T_from(SU)to(IM)_)_*period(2,3)_	*652.02*
M7	P_(constant)_,S_(SU or IM or INF)_, T_from(SU,IM,INF)to(SU,IM,INF)_	661.49

Model id	2006/models	QAICc
M8	P_(trap site)_,S_(SU,IM)_,T_from(SU,IM)to(SU,IM)*period(1,2,3)_	1859.55
M9	P_(trap site)_,S_(SU or IM)_,T_from(SU,IM)to(SU,IM)*period(1,2,3)_	1890.13
*M10*	P_(trap site)_,S_(SU,IM)_, T_from(SU)to(IM)+_ T_from(IM)to(SU)*period(1,2,3)_	*1859.76*
M11	P_(trap site)_,S_(SU,IM)_, T_from(SU,IM)to(SU,IM)_	1893.94

P corresponds to the probability of recapture, S to the survival and T to the transition probabilities between the disease states (SU, IM, INF). The selected model for each year is in *italic (M6 and M10)*.

### 5. Model predictions

In 2005, infections were observed during the entire capture period. We estimated that the average duration of infection was 1.18 weeks and that proportions of lethal-chronic, lethal-acute and transient disease courses were: P_chronic_ = 0.001, P_acute_ = 0.795, P_transient_ = 0.204. In 2006, we detected no infected piglets but we cannot dismiss unobserved infections since animals acquired antibodies out of the vaccination period (i.e., period 2) and since the survival rate was lower in susceptible than in immune piglets.

## Discussion

Our longitudinal study of individual survival/infection histories showed that CSF was highly lethal and vaccination ineffective in piglets.

During the study, most of the infected piglets (80%) did not survive more than two weeks, while the others (20%) quickly recovered, and were thus transiently (i.e., briefly) infected. Even though we cannot rule out a rare occurrence of chronic infection, our study demonstrates that chronic infection seldom occurs among wild piglets. This result is contrary to the previous observation [13] of infected piglets surviving 39 days. However, this former study was conducted in a single piglet litter and under laboratory conditions, which may have enhanced artificially the survival of infected individuals. According to the models developed by Kramer-Schadt et al. [44], a virus being so lethal in piglets in the wild is unlikely to persist by circulating only in that age class. We thus consider that piglets did not constitute the main CSF reservoir, even though the proportion of infected individuals observed in the hunting bags was higher in young animals than in adults [17]. Alternatively, we hypothesize that chronic infections occurred more frequently in older animals, which are more resistant than piglets to the pathogenic action of CSF [32], even though these individuals have been difficult to detect using the hunting data [11], [17]. Unfortunately, we could not test this hypothesis given that older animals are very difficult to recapture weekly. We also have to consider that the population size (conditioned by the size of the forested areas) is an important factor for disease persistence since the probability of maintaining the chain of transmission through chronic infections increases with the number of animals [7], [44]. In a large forest (ex: Vosges Mountains and Palatinate), CSF might persist and spread again despite infection being extinct in a given locality (ex: PPNR). It is thus important that management measures for controlling CSF are implemented to the whole area at risk [17].

For piglets, the probability of becoming immune to CSF appeared to be unrelated to vaccination, whatever the vaccination period. Indeed, the probability of becoming immune did not increase during the summer vaccination sessions in both years of the study, and the proportion of immune piglets was similar among those hunted in early winter, after the autumn vaccination sessions (September), and among those captured in late August. These results suggest a low efficacy of the two first vaccination sessions in piglets. This result may arise during the summer because piglets were too small to eat the baits [15]. The age of piglets had been considered as the main factor driving their capacity to eat the vaccine-baits because in captivity consumption had been observed only among the piglets that were more than 4.5 months old [15]. But in the present study we did not detect an effective immunization of piglets in autumn, i.e., when most piglets were 6–7 months old. We thus consider that the age of piglets was not the only factor that influenced the vaccine-bait uptake during the study. A competition with alternative food sources such as crops and oak mast may have also decreased the palatability of baits to wild boar [31]. Although ineffective in summer and autumn, vaccinating piglets in this area seems possible during wintertime [31], i.e., when most animals are large enough to eat baits and when the food availability does not compete with the vaccine baits. Since 2007, the autumn sessions have been moved from September to November or December [17]. New baits have been recently developed to try to vaccinate piglets in their early life for a better control of CSF [15] or bovine tuberculosis [48]. However, given that alternative food sources cannot be avoided [31] and that animals more than 6 months old are possibly more likely to maintain the chain of transmission than piglets (results of the present study), to improve vaccination in wintertime is possibly the best option for improving CSF control in this European eco-region.

Our capture-mark-recapture approach was useful for assessing individual disease outcome and vaccination effect. By considering the effect of the trap-site in the recapture-probability and by removing the first capture from each individual history, we avoided the major infringement of the model hypotheses. However, we cannot exclude some biases in the CMR process. First, our trapping was certainly biased in favour of the social groups having a dominant status on the feeding grounds and thus being more likely to be vaccinated than others (baits are delivered on the feeding grounds) [31], [49]. Secondly, the accuracy of model estimations may have been limited because we did not capture all the animals every week and we could have missed short-term infections between two consecutive recaptures. Moreover, false negative or positive results can never be excluded [50]. In particular, it is likely that a fluctuation in the serological results when maternal derived antibodies became low has generated part of the flux we observed between the susceptible and the immune states outside of the vaccination periods. However, we consider that these methodological limitations did not invalidate our qualitative interpretation of the individual histories and main results: i.e., the short and lethal infections in piglets, the low efficacy of vaccination. While the former studies based on hunting data only hypothesized the role of piglets from average percentages, the multi-state recapture approach used here explored the true kinetics of infection and the effect of vaccination in the wild. This study has thus clarified the role of piglets (minor) and the factors influencing vaccination efficacy (i.e., the food availability and not only the age of piglets). We finally recommend this approach for a better understanding of wildlife diseases when capture-mark-recapture data are available and may complete the cross-sectional surveys [51].
